# An Efficient Object Tracking Method on Quad-/Oc-Trees

**DOI:** 10.1371/journal.pone.0150889

**Published:** 2016-03-17

**Authors:** Magda Przybylowski, Pratim Ghosh, Frederic Gibou

**Affiliations:** 1 Department of Computer Science, University of California Santa Barbara, Santa Barbara, CA 93106-5110, United States of America; 2 Department of Mechanical Engineering, University of California Santa Barbara, Santa Barbara, CA 93106-5070, United States of America; 3 Department of Electrical and Computer Engineering, University of California Santa Barbara, Santa Barbara, CA 93106-5110, United States of America; University of Akron, UNITED STATES

## Abstract

We introduce a fast error-free tracking method applicable to sequences of two and three dimensional images. The core idea is to use Quadtree (resp. Octree) data structures for representing the spatial discretization of an image in two (resp. three) spatial dimensions. This representation enables one to merge into large computational cells the regions that can be faithfully described with such a coarse representation, thus significantly reducing the total number of degrees of freedom that are processed, without compromising accuracy. This encoding is particularly effective in the case of algorithms based on moving fronts, since the adaptive refinement provides a natural means to focus the processing resources on information near the moving front. In this paper, we use an existing contour based tracker and reformulate it to the case of Quad-/Oc-tree data structures. Relevant mathematical assumptions and derivations are presented for this purpose. We then demonstrate that, on standard bio-medical image sequences, a speed up of 5*X* is easily achieved in 2D and about 10*X* in 3D.

## 1 Introduction

Tracking objects in image sequences is a fundamental problem in computer vision. Error-free tracking is essential for various vision applications such as surveillance, security systems, and medical/biological image analysis. However, data uncertainty presents significant challenges for reliable object tracking. This includes, for example, illumination changes across consecutive frames, rigid/nonrigid deformation of the object of interest, partial occlusion, or data corruption.

Numerous frameworks have been proposed in recent years to address the above-mentioned problems. For example, a class of methods includes deformable shape models [1], active shape models (ASMs) [2] and active appearance models (AAMs) [3], which capture the statistics of shape and appearance variations using training examples. By combining ASM and AAM in a multiscale fashion, Mitchell *et al.* [4] achieved robustness against noise and clutter. Tsai *et al.* [5] applied principal component analysis (PCA) to model the variability in the training shapes represented by signed distance functions (SDFs). Zhu *et al.* [6] proposed a subject-specific dynamic model designed for medical application using multilinear PCA. A potential drawback of these methods is that their performance depends on the training data, which may not encompass all possible scenarios.

Recently, graph-based algorithms have been proposed for tracking deformable objects. For example, Ishikawa and Jermyn [7] developed a polynomial time algorithm to extract similar objects from a set of time sequence images. Schoenemann and Cremers [8] proposed a real-time solution for tracking, implemented on GPUs, that is based on finding a minimum cost cyclic path in the product space spanned by the template shape and the given image. The cost function is derived from both the image data and the shape prior. They then extended their previous approach [9] by incorporating the edge information, which provides robustness against illumination changes. In another work [10], they also introduced a motion layer decomposition framework, which is solved using both discrete and continuous optimization. Although this method has been shown to be robust against occlusion, it is unclear how this algorithm may be adapted for tracking objects with unknown shape statistics. Discriminative methods [11, 12], which cast visual tracking as a two-class classification problem, dynamically update the classifiers in order to account for appearance changes and partial occlusion of the object-of-interest. Approaches such as [13] select the target as the one which minimizes the projection error in the space spanned by observed (tracked results from previous frames) and trivial templates (with one nonzero element).

Rich, dense flow information between consecutive frames in time sequence images can also be incorporated as a means to improve effectiveness. One way to include this information is to first compute a correspondence map (registration) between successive frames before computing the correlated segmentation of the video frames to solve the tracking problem. In recent works [14, 15], it has been shown that tracking performance can be further improved by solving the registration and the segmentation problems simultaneously (SRS for simultaneous registration and segmentation) thanks to a function that establishes the correspondence between the target and the reference level-set functions. The performance is improved in [16] with the help of a dynamical prior term (SRS+DP). The same authors recently generalized the SRS+DP framework (GSRS for generalized SRS), which can handle shading, reflections, and illumination changes. In [17], Ghosh *et al.* extend GSRS [18] for fast and efficient implementation. Modifications include the reconstruction of the sparse-error (due to partial occlusion, shading and reflections) between consecutive frames using a regularized variant of the *L*^1^ norm, a new formulation of the dynamical shape prior term and the incorporation of a combination term, which modifies the update rule for the estimated functions. Through examples with natural and biological images sequences, the authors have illustrated the robustness of their formulation.

However, another fundamental problem in computer vision is the efficiency of the underlying strategy, in terms of CPU and memory requirement. A fast error-free tracking can be determining in some applications such as medical analysis or security systems. Medical analysis, with the ever increasing amount of data generated by modern acquisition systems, is in particular an area where large data sets need to be processed efficiently. The effectiveness of the approach of Ghosh *et al.* [17] is in large part due to the use of rich dense flow information between consecutive frames, a computationally expensive feature. However, dense flow information is mainly useful in the region near the evolving front so that the use of adaptive grid techniques that automatically coarsen away from the moving front have a distinct advantage. The key contribution of this paper is the use of Quad-/Oc-tree Cartesian grids for efficient processing of an algorithm that is robust against partial occlusion, drastic illumination changes, and data corruption.

The outline of this paper is as follows: we first briefly introduce the level-set formalism in section 2 and the SRS method with sparse error reconstruction in section 3. In section 4, we develop the algorithm on Quad-/Oc-tree Cartesian grids. We finally compare the results obtained with the present method and that of [17] in section 5.

## 2 Level-Set Formalism

The level-set method, introduced by Osher and Sethian [19], is a general numerical technique for capturing the evolution of a moving front. Specifically, the front is represented as the zero level set of Lipschitz continuous function *ϕ*. We will denote the moving front as Γ and thus define Γ = {**x** : *ϕ*(**x**) = 0}. The interior and exterior regions delimited by Γ are described by {**x** : *ϕ*(**x**) > 0} and {**x** : *ϕ*(**x**) < 0}, respectively. The implicit level-set representation is particularly convenient in the case of topological changes. Given a velocity field **u**, the motion of the front is given by the equation:

$$\phi_{t}+u\cdot \nabla \phi =0.$$

Finally, while any Lipschitz continuous function can define Γ, it is advantageous to use the signed distance function to the front. For a general front described by an arbitrary level-set function *ϕ*_init_, the signed distance function is obtained by solving, in pseudo time *τ*, the equation:

$$\phi_{\tau }+\text{Sign}\left(\phi_{\text{init}}\right)\left(|\nabla \phi |-1\right)=0,$$

where Sign refers to the signum function. This is the so-called reinitialization equation, introduced for uniform grids by Sussman *et al.* [20], improved by Russo and Smereka [21] and extended to unstructured grids in Min and Gibou [22].

## 3 Simultaneous Registration and Segmentation

Consider two consecutive images $I\left(t-1\right):\Omega \left(\subseteq R^{n}\right)\to R$, with *n* = 2, 3 and $I\left(t\right):\Omega \left(\subseteq R^{n}\right)\to R$, in an image sequence at two consecutive time instances, *t* − 1 and *t*. Assuming that the initial contour of the object of interest is known *a priori*, we consider that the shape of the tracked object of interest at any arbitrary time *t* is embedded in the level-set function $\Phi^{0}\left(x,t\right):\Omega \to R^{n}$.

### 3.1 Registration Model

The aim of registration is to find a displacement vector field $u:\Omega \to R^{n}$ that establishes the dense correspondence between two consecutive image frames. We denote the components of **u** as (*u*(**x**), *v*(**x**)) and (*u*(**x**), *v*(**x**), *w*(**x**)) in two and three spatial dimensions, respectively. This vector field is traditionally obtained by maximizing the posterior distribution *P*(**u**|*I*(*t*), *I*(*t* − 1)) (see for example [1, 23] and references therein):

$$\begin{array}{ccc} u^{*} & = & \underset{u}{\mathrm{argmax}}\;P\left(u|I\left(t\right),I\left(t-1\right)\right)\\ & ∼ & \underset{u}{\mathrm{argmax}}\;P\left(I\left(t\right),I\left(t-1\right)|u\right)\;P\left(u\right). \end{array}$$ (1)

In the present work, based on [17], we simultaneously estimate both misalignment (dense correspondence map) and corruption in the image sequence by assuming that the corruption due to partial occlusion, shading, reflections, etc., is sparse in nature. Consider that the mapping G:Ω→R models the sparse corruptions between successive time frames. We can rewrite Eq (1) by incorporating *G* as follows:

$$\begin{array}{ccc} <u^{*},G^{*}> & = & \underset{u,G}{\mathrm{argmax}}\;P\left(u,G|I\left(t\right),I\left(t-1\right)\right)\\ & ∼ & \underset{u,G}{\mathrm{argmax}}\;P\left(I\left(t\right),I\left(t-1\right)|u,G\right)P\left(u,G\right)\\ & = & \underset{u,G}{\mathrm{argmax}}\;P\left(I\left(t\right),I\left(t-1\right)|u,G\right)P\left(u\right)P\left(G\right), \end{array}$$ (2)

where we assumed in the last step that **u** and *G* are independent of each other. Maximizing the expression in Eq (2) is equivalent to minimizing the following energy functional under certain simplifying assumptions:

$$\begin{array}{ccc} \min_{u,G}E_{RG}\left(u,G;I\left(t-1\right),I\left(t\right)\right) & = & \int_{\Omega }\rho_{u}{\left(|I\left(T\left(x\right),t\right)-I\left(x,t\;-\;1\right)-G\right|}^{2})dx\\ & + & \alpha_{u}\int_{\Omega }\rho_{u}{\left(|\nabla u\right|}^{2}+{\left|\nabla v\right|}^{2})dx\\ & + & \alpha_{g_{1}}\int_{\Omega }\rho_{u}{\left(|G\right|}^{2})dx+\alpha_{g_{2}}\int_{\Omega }\rho_{u}{\left(|\nabla G\right|}^{2})dx, \end{array}$$ (3)

where, **T**(**x**) **=** **x** + **u**(**x**), *α*_*u*_ controls the smoothness of the derived vector field, while *α*_*g*_1__ and *α*_*g*_2__ control the sparsity and the smoothness in the estimated corruption. It has been demonstrated in [3] that *L*^1^-minimization is more suitable than the quadratic penalizer, *ρ*(*x*^2^) = *x*^2^; hence we use $\rho_{u}=\sqrt{x^{2}+\epsilon^{2}}$, the most robust convex penalizer known in the optical flow community. Due to the small positive constant *ϵ*, *ρ*_*u*_ is still convex, which offers advantages for the minimization process. The constant *ϵ* is chosen in our experiments to be 0.001.

Since the energy *E*_*RG*_ is highly nonlinear, the minimization process is not trivial. A minimizer for **u** must satisfy the Euler-Lagrange equations:

$$\begin{array}{ccc} C_{u} & = & \rho_{u}^{\prime }\left(I_{d}^{2}\right)I_{d}I_{x}-\alpha_{u}\nabla \cdot (\rho_{u}^{\prime }{\left(|\nabla u\right|}^{2}+{\left|\nabla v\right|}^{2})\nabla u)=0,\\ C_{v} & = & \rho_{u}^{\prime }\left(I_{d}^{2}\right)I_{d}I_{x}-\alpha_{v}\nabla \cdot (\rho_{u}^{\prime }{\left(|\nabla u\right|}^{2}+{\left|\nabla v\right|}^{2})\nabla v)=0, \end{array}$$ (4)

where

$$\begin{array}{ccc} I_{d} & = & I(T(x),t)-I(x,t-1)-G,\\ I_{x} & = & \partial_{x}I\left(T\left(x\right),t\right),\\ I_{y} & = & \partial_{y}I\left(T\left(x\right),t\right). \end{array}$$

Similarly, the Euler-Lagrange equation that needs to be satisfied by *G* is:

$$\begin{array}{ccc} -\rho_{u}^{\prime }\left(I_{d}^{2}\right)\;I_{d}I_{x}+\alpha_{g_{1}}\rho_{u}^{\prime }{\left(|G\right|}^{2})\;G-\alpha_{g_{2}}\nabla \cdot (\rho_{u}^{\prime }{\left(|\nabla G\right|}^{2})\nabla G) & = & 0. \end{array}$$

To solve these equations, we adapted the approach from [24, 25]: **u** and *G* are both solved iteratively in multiple scales using fixed point schemes. We implement a fully implicit scheme for the smoothing terms (e.g., the term associated with *α*_*u*_ and *α*_*g*2_) and a semi-implicit scheme for the data terms (e.g., the first term in Eq (3) and the term associated with *α*_*g*1_). The term corresponding to warping, *I*(**T**(**x**), *t*), is obtained using linear interpolation.

### 3.2 Segmentation Model

It is standard to segment the *t*-frame using the dynamic prior term, given the observed contour *ϕ*^0^(*t*). However, there are a few drawbacks with this approach. Examples include the absence of an energy minimizer or the dependence on the choice of the domain. These can be avoided with the formulation of Ghosh *et al.* [17], which is based on the dual formulation of the total variation (TV) norm, by including the shape prior term to define a new dynamic shape prior term, $d_{p}^{2}:\Omega \to R^{+}$:

$$\begin{array}{c} d_{p}^{2}\left(\phi^{o}\left(x\right);{\hat{\phi }}^{-}\left(x\right)\right)=\int_{\Omega }\rho_{p}\left(\right|{\hat{\phi }}^{-}{\left(x\right)|}^{2})|\nabla H\left(\phi^{o}\right)|dx, \end{array}$$ (5)

where *ρ*_*p*_(.) is a robust estimator; here we assume *ρ*_*p*_(*y*^2^) = *y*^2^. One then considers the following constrained minimization problem:

$$\begin{array}{ccc} \min_{0\leq \phi^{0}\leq 1}E_{DS}\left(\phi^{0}\left(x\right);I\left(t\right),d_{p}^{2}\right) & = & \underset{TV_{d_{p}^{2}}\left(\phi^{0}\right)}{\underset{︸}{\int_{\Omega }d_{p}^{2}\left|\nabla \phi^{0}\left(x\right)\right|dx}}+\alpha_{s_{1}}\int_{\Omega }\underset{\eta (x,\theta_{1},\theta_{2})}{\underset{︸}{\ln(\frac{p(I|\theta_{2})}{p(I|\theta_{1})}}}\phi^{0}\left(x\right)dx. \end{array}$$ (6)

The solution is constrained to lie between 0 and 1 since the unconstrained problem does not have a stationary solution. The first term in Eq (6) measures the weighted total variation norm of the function *ϕ*^0^. It can be rigorously demonstrated (see [26, 27] and references therein) that Eq (6) is equivalent to solving the following unconstrained problem:

$$\begin{array}{ccc} \min_{0\leq \phi^{0}\leq 1}E_{DS}\left(\phi^{0}\left(x\right);I\left(t\right),d_{p}^{2}\right) & = & TV_{d_{p}^{2}}\left(\phi^{0}\right)+\int_{\Omega }\left[\alpha_{s1}\eta \left(x,\theta_{1},\theta_{2}\right)\phi^{0}+\hat{\alpha }\zeta \left(\phi^{0}\right)\right]dx, \end{array}$$ (7)

where $\zeta \left(y\right):=\max\left\{0,2|y-\frac{1}{2}|-1\right\}$ and $\hat{\alpha }>\frac{\alpha_{s1}}{2}{\left||\eta \left(x\right)|\right|}_{L^{\infty }\left(\Omega \right)}$. We can also rewrite *ζ*(*y*) as:

$$\zeta \left(y\right)=\left\{\begin{array}{cc} -2y & \text{if}\;\;y<0,\\ 2(y-1) & \text{if}\;\;y>1,\\ 0 & \text{otherwise}. \end{array}\right.$$

Since the two energies in Eqs (6) and (7) agree for {*ϕ*^0^ ∈ *L*^∞^(Ω) : 0 ≤ *ϕ*^0^(**x**) ≤ 1 ,∀**x**} it is sufficient to prove that any minimizer of Eq (7) satisfies the constraint 0 ≤ *ϕ*^0^(**x**) ≤ 1. The variational formulation (7) can now be solved using a convex regularization [28] term:

$$\begin{array}{c} \underset{\phi^{o},\varphi }{\text{min}}E_{DS}(\phi^{o},\varphi ;I(t),d_{p}^{2})\\ \;=\text{T}{\text{V}}_{d_{p}^{2}}(\phi^{o})+\frac{1}{2\beta }||\phi^{o}-\varphi |{|}^{2}\\ \;+\underset{\Omega }{\int }[\alpha_{s_{1}}\eta (x,\theta_{1},\theta_{2})\varphi +\alpha \zeta (\varphi )]dx, \end{array}$$ (8)

where *β* > 0 is a small constant, so that *φ* is a close approximation of *ϕ*^*o*^.

### 3.3 Simultaneous Registration and Segmentation

A commonly used objective function in this regard is the one which establishes the correspondence between the target (*ϕ*^*o*^(*t*)) and the reference (*ϕ*^*o*^(*t* − 1)) level-set functions using a mapping function **u**(**x**, *t* − 1, *t*). The functional combination term seeks to register the isocontours of the source and target shapes within a narrow band of the zero level sets. The width of the narrow band is chosen by a parameter *ϵ*′. The choice of *ϵ*′ is crucial since it determines the scale at which objects are registered (see [29] for a detailed discussion). In contrast, Ghosh *et al.* [17] proposed a functional that does not require the scale information *a priori*. In this case, the combination term is (similar to Eq (5)) defined as:

$$\begin{array}{ccc} \min_{u,\phi^{o}\left(t\right)}\;E_{NC}(u & ,\phi^{o}\left(t\right);\phi^{o}\left(t-1\right))=d_{c}^{2}= & \int_{\Omega }\rho_{c}\left(\right|\phi^{o}{\left(T\left(x\right),t-1\right)|}^{2})|\nabla H\left(\phi^{o}\left(x,t\right)\right)|dx, \end{array}$$

where *ρ*_*c*_(*y*^2^) = *y*^2^. Finally, the method simultaneously estimates **u**, G, and *ϕ*^0^(*t*) for the current frame at time *t*. This combination term modifies the update rule for the estimated functions. Recalling Eq (4), the new Euler-Lagrange equations that need to be satisfied for **u** can be written as:

$$\begin{array}{ccc} \rho_{c}^{\prime }\left(\phi_{\omega }^{2}\right)\phi_{\omega }\phi_{x}|\nabla H(\phi^{0}\left(t\right)|+C_{u} & = & 0,\\ \rho_{c}^{\prime }\left(\phi_{\omega }^{2}\right)\phi_{\omega }\phi_{y}|\nabla H(\phi^{0}\left(t\right)|+C_{w} & = & 0, \end{array}$$

where

$$\begin{array}{c} \phi_{\omega }=\phi^{0}\left(T\left(x\right),t-1\right),\;\phi_{x}=\partial_{x}\phi_{\omega }\;\text{and}\;\phi_{y}=\partial_{y}\phi_{\omega }. \end{array}$$

The update rule for the sparse error, *G*(*x*, *y*), remains the same since it is independent of the combination term *E*_*NC*_. For the segmentation problem, we use:

$$\begin{array}{c} \min_{\phi^{0}}\{TV_{d_{p}^{2}+d_{c}^{2}}\left(\phi^{0}\right)+\frac{1}{2\beta }\left|\right|\phi^{0}-{\varphi ||}^{2}\}. \end{array}$$ (9)

This formulation has been shown to be robust against partial occlusion, drastic illumination changes, and data corruption. A drawback of the existing approach is the computational cost associated with solving the partial differential equations of the model on a grid with as many degree of freedom as the number of pixels/voxels. However, the region of interest for processing the data at every step is located in only a small band around the moving front. It is therefore desirable to adopt a strategy that can leverage the locality of information. Next, we describe an algorithm based on Quad-/Oc-tree data structure, which enables to significantly decrease the number of degrees of freedom, while conserving the accuracy of the method of [17].

## 4 Algorithm on Quad/Oc-tree Data Structures

In this section, we describe how the level-set method on Quad-/Oc-tree introduced by Min and Gibou [22] can be combined with the methodology of [17] to produce an efficient algorithm for object tracking and segmentation.

### 4.1 Quad/Oc-tree Data Structures

**Fig 1:**
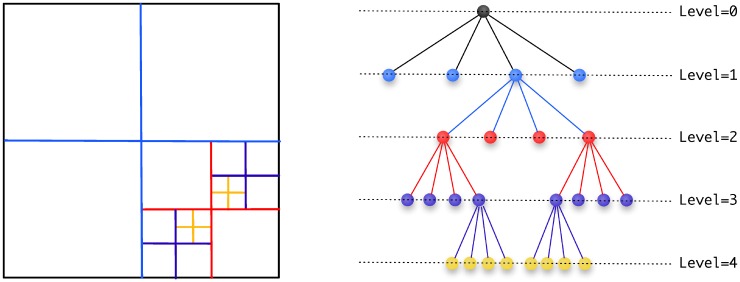
Discretization of a two-dimensional domain (left) and its quadtree representation (right). The entire domain corresponds to the root of the tree (level 0). Each cell can then be recursively subdivided further into four children. In this example, the tree is non-graded, since the difference of level between some adjacent cells exceeds one. (Color online.)

Quadtree (resp. Octree) data structures can be used to represent the spatial discretization of a geometrical domain in two (resp. three) spatial dimensions as depicted in Fig 1: initially the root of the tree is associated with the entire domain cell. Further refinement is obtained by recursively splitting each cell into four (resp. eight) children until the desired level of detail is achieved. We call ‘level of the tree’ its depth. We refer the reader to the books of Samet [30, 31] for more details on the definition of these data structures and the standard algorithms associated with them.

In this work, the maximum level of the tree is that corresponding to the pixel size, i.e. a 1024 × 1024 image will be associated with a quadtree with a maximum level of 10 (as in 2^10^). Also, in the case where a tree cell is associated to a subset of an image, the cell’s value is defined as the average pixels’ value.

### 4.2 Constructing Quad-/Oc-tree

Since the accuracy of the method depends on the size of the cells adjacent to the moving front, we impose that the finest cells lie on the interface. This can be achieved by using the signed distance function to the interface along with the Whitney decomposition, as first proposed by Strain in [32]. For a general function $\phi :R^{n}\to R$ with Lipschitz constant Lip(*ϕ*), the Whitney decomposition is extended in Min [22] as: starting from a root cell, split any cell C if

$$\begin{array}{c} \max_{v\in \text{Nodes}(C)}\left|\phi \left(v\right)\right|\leq \frac{1}{2}Lip\left(\phi \right)\times diag-size\left(C\right), \end{array}$$

where diag-size(*C*) refers to the length of the diagonal of the current cell *C* and Nodes(*C*) refers to the set of four (resp. eight) nodes of the current cell. This condition enforces the splitting of any cell whose edge length exceeds its distance to the interface, hence producing a computational grid where the smallest cells capture the interface location. Larger and larger cells are located as the distance from the interface increases (see [22] for details).

**Fig 2:**
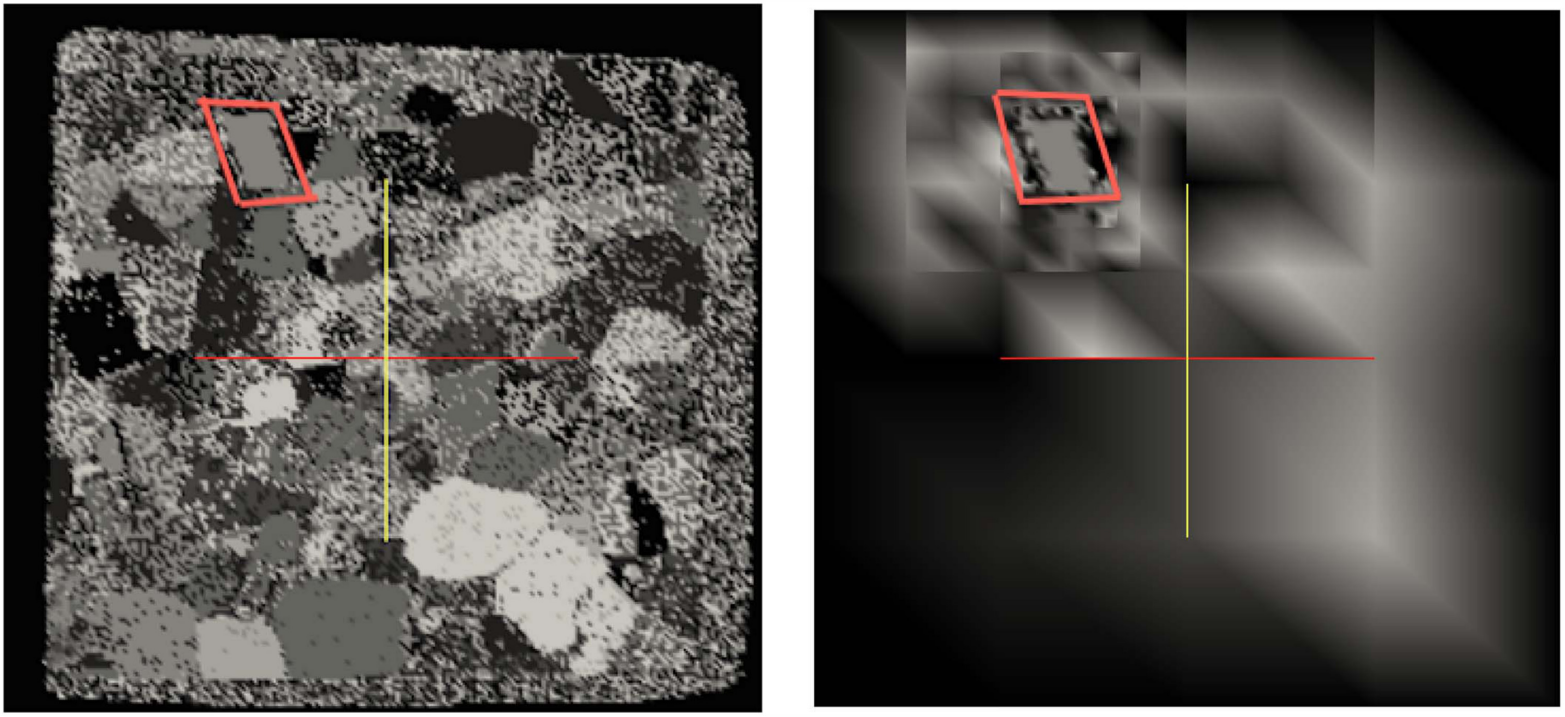
Quadtree refinement of an image.

Fig 2 depicts an example of an image (left) and its quadtree representation (right). Note that far from the simulated moving front (depicted by a red interface), the image is blurred due to averaging of pixels’ value, while the original image resolution is kept near the interface. In the work of [16], the image was filtered by a gaussian filter to avoid spurious noise. In the case of Quad-/Oc-tree representation, the coarsening away from the front acts as a filter so we do not apply a Gaussian filter.

### 4.3 Finite Difference Discretizations

**Fig 3:**
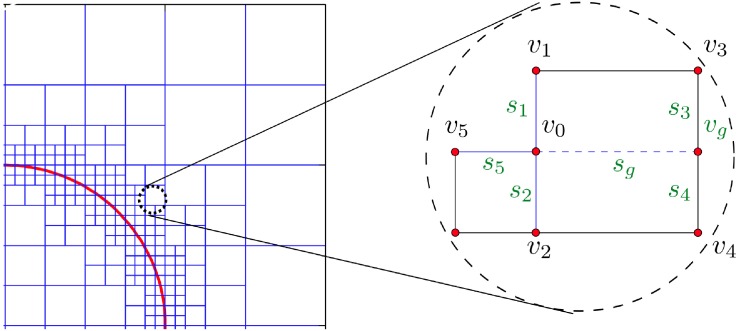
Local grid configuration near a node *v*0. The schematic on the right describes a T-junction where a node is missing in the *x*-direction.

In the case of nonregular Cartesian grids, the main difficulty is to derive discretizations at T-junction nodes, i.e. nodes for which there is a missing neighboring node in one of the Cartesian directions. For example, Fig 3 depicts a T-junction node, *v*_0_, with three neighboring nodes *v*_1_, *v*_2_ and *v*_5_ aligned in three Cartesian directions and one ghost neighboring node, *v*_*g*_, replacing the missing grid node in the *x*-direction. Referring to Fig 3, the value, $u_{g}^{G}$, of the node-sampled function $u:\{v_{i}\}\to R$ at the ghost node *v*_*g*_ is given by the third-order interpolation:

$$u_{g}^{G}=\frac{u_{3}s_{4}+u_{4}s_{3}}{s_{3}+s_{4}}-\frac{s_{3}s_{4}}{s_{1}+s_{2}}\left(\frac{u_{1}-u_{0}}{s_{1}}+\frac{u_{2}-u_{0}}{s_{2}}\right).$$

**Fig 4:**
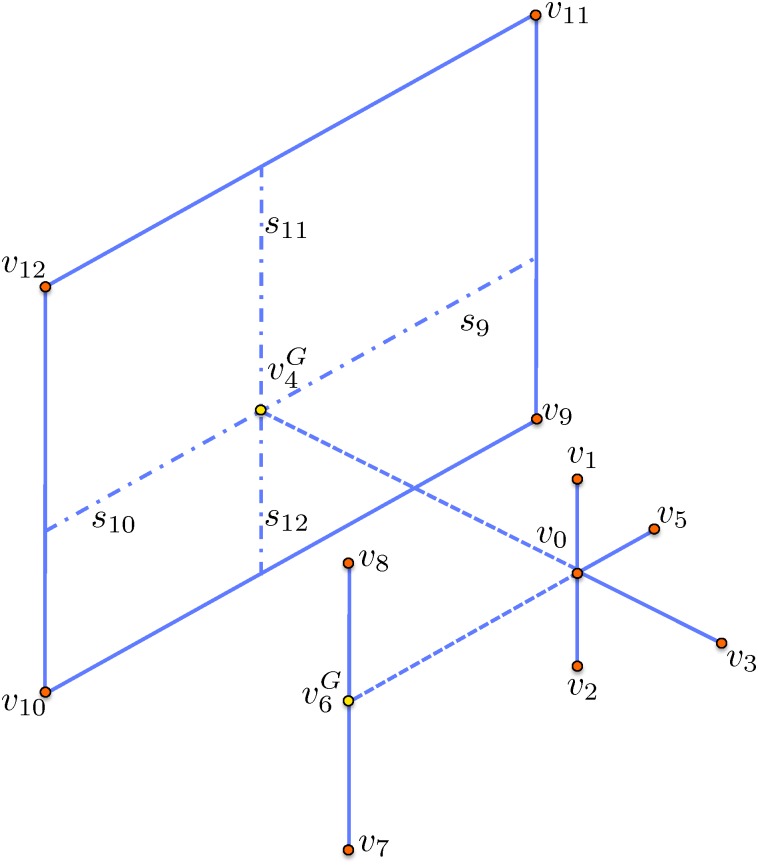
Neighboring vertices of a node *v*_0_ in three spatial dimensions.

In three spatial dimensions, there are two ghost values (see Fig 4) that are defined as [22]:

$$\begin{array}{cc} u_{6}^{G} & =\frac{s_{8}u_{7}+s_{7}u_{8}}{s_{8}+s_{7}}-\frac{s_{8}s_{7}}{s_{2}+s_{1}}\left(\frac{u_{2}-u_{0}}{s_{2}}+\frac{u_{1}-u_{0}}{s_{1}}\right),\\ u_{4}^{G} & =\frac{s_{11}s_{12}u_{11}+s_{11}s_{9}u_{12}+s_{10}s_{12}u_{9}+s_{10}s_{9}u_{10}}{\left(s_{10}+s_{11}\right)\left(s_{9}+s_{12}\right)}\\ & \;-\frac{s_{10}s_{11}}{s_{2}+s_{1}}\left(\frac{u_{2}-u_{0}}{s_{2}}+\frac{u_{1}-u_{0}}{s_{1}}\right)\\ & \;-\frac{s_{9}s_{12}}{s_{1}+s_{4}}\left(\frac{u_{1}-u_{0}}{s_{1}}+\frac{u_{6}^{G}-u_{0}}{s_{4}}\right). \end{array}$$

**Fig 5:**
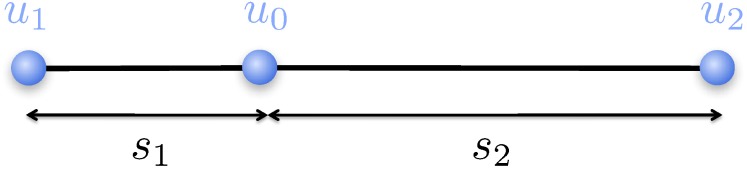
A one-dimensional adaptive grid.

The third-order interpolations defined above allow us to define finite difference formulas for the first- and second-order derivatives at every node using standard formulas in a dimension-by-dimension framework. For example, referring to Fig 5, we use the central difference formulas for *u*_*x*_ and *u*_*xx*_:

$$D_{x}^{0}u_{0}=\frac{u_{2}-u_{0}}{s_{2}}\cdot \frac{s_{1}}{s_{1}+s_{2}}+\frac{u_{0}-u_{1}}{s_{1}}\cdot \frac{s_{2}}{s_{1}+s_{2}},$$

$$D_{xx}^{0}u_{0}=\frac{u_{2}-u_{0}}{s_{2}}\cdot \frac{2}{s_{1}+s_{2}}-\frac{u_{0}-u_{1}}{s_{1}}\cdot \frac{2}{s_{1}+s_{2}},$$

the forward and backward first-order accurate approximations of the first-order derivatives:

$$\begin{array}{cc} D_{x}^{+}u_{0} & =\frac{u_{2}-u_{0}}{s_{2}},\\ D_{x}^{-}u_{0} & =\frac{u_{0}-u_{1}}{s_{1}}, \end{array}$$

and the second-order accurate approximations of the first-order derivatives:

$$\begin{array}{cc} D_{x}^{+}u_{0} & =\frac{u_{2}-u_{0}}{s_{2}}-\frac{s_{2}}{2}\text{minmod}\left(D_{xx}^{0}u_{0},D_{xx}^{0}u_{2}\right),\\ D_{x}^{-}u_{0} & =\frac{u_{0}-u_{1}}{s_{1}}+\frac{s_{1}}{2}\text{minmod}\left(D_{xx}^{0}u_{0},D_{xx}^{0}u_{1}\right), \end{array}$$

where the minmod slope limiter [33, 34], defined as:

$$\text{minmod}\left(x,y\right)=\{\begin{array}{cc} x & \text{if}\left|x\right|>\left|y\right|,\\ y & \text{otherwise}, \end{array}$$

is used to avoid differencing across regions where gradients are large (i.e. near kinks). Similarly, approximations for first-order and second-order derivatives are obtained in the *y*- and *z*- directions. These numerical approximations are sufficient to discretize all of the equations described in section 3.

## 5 Experimental Results

In this section, we first present results on two and three dimensional image sequences. When available, we compare our results to those obtained in Ghosh *et al.* [16], which allows us to compare the effectiveness of both methods in term of robustness as well as in terms of computational efficiency. These numerical examples illustrate that the present method is robust even in the presence of severe occlusions and provides a speed up over the approach of Ghosh *et al.* [16]. The advantage of the present method is that it significantly reduces the number of degrees of freedom processed at each iteration. In fact, since most degrees of freedom are those adjacent to the interface, this approach lowers the dimension of the problem. On the other hand, there are additional costs associated to the handling of the data structure, especially the *O*(log(*n*)) cost to access data stored in the leaves of the tree and the remeshing procedure. Despite this additional cost, the performance of the current approach is superior than that on uniform grids.

### 5.1 Parameter Setting

We keep all the parameters constant throughout our experiments. The parameters *α*_*u*_, *α*_*g*_1__, and *α*_*g*_2__ are empirically set to 8 × 10^−4^, 9 × 10^−5^, and 3 × 10^−3^, respectively. The weight for the dynamical prior term $d_{p}^{2}$ and the combination term $d_{c}^{2}$ are both approximately set to 2 × 10^−5^. The coefficient *α*_*s*_1__ corresponding to Eq (8) is selected randomly in the range [0.02, 0.05].

We first extract a simple gray scale histogram (for both natural and synthetic images) of the foreground and background from a few frames. These are then used to obtain the likelihood values *η*(*x*) for the rest of the frames. In the current implementation, we also use the edge information of the image. The edge information is easily incorporated by minimizing $TV_{g+d_{p}^{2}+d_{c}^{2}}$ instead of $TV_{d_{p}^{2}+d_{c}^{2}}$ in Eq (9). Here, $g=\frac{1}{1+\gamma |\nabla I_{\sigma }{|}^{2}}$ is the inverse of the smoothed gradient magnitude function. In our experiments, we keep *γ* = 600.

The proposed tracking framework is implemented in C++ on a 2.3 GHz Intel Core i5 CPU. In our implementation we update **u**, *ϕ*^0^, and *G* twice for each time frame.

### 5.2 Two Dimensional Data

**Fig 6:**
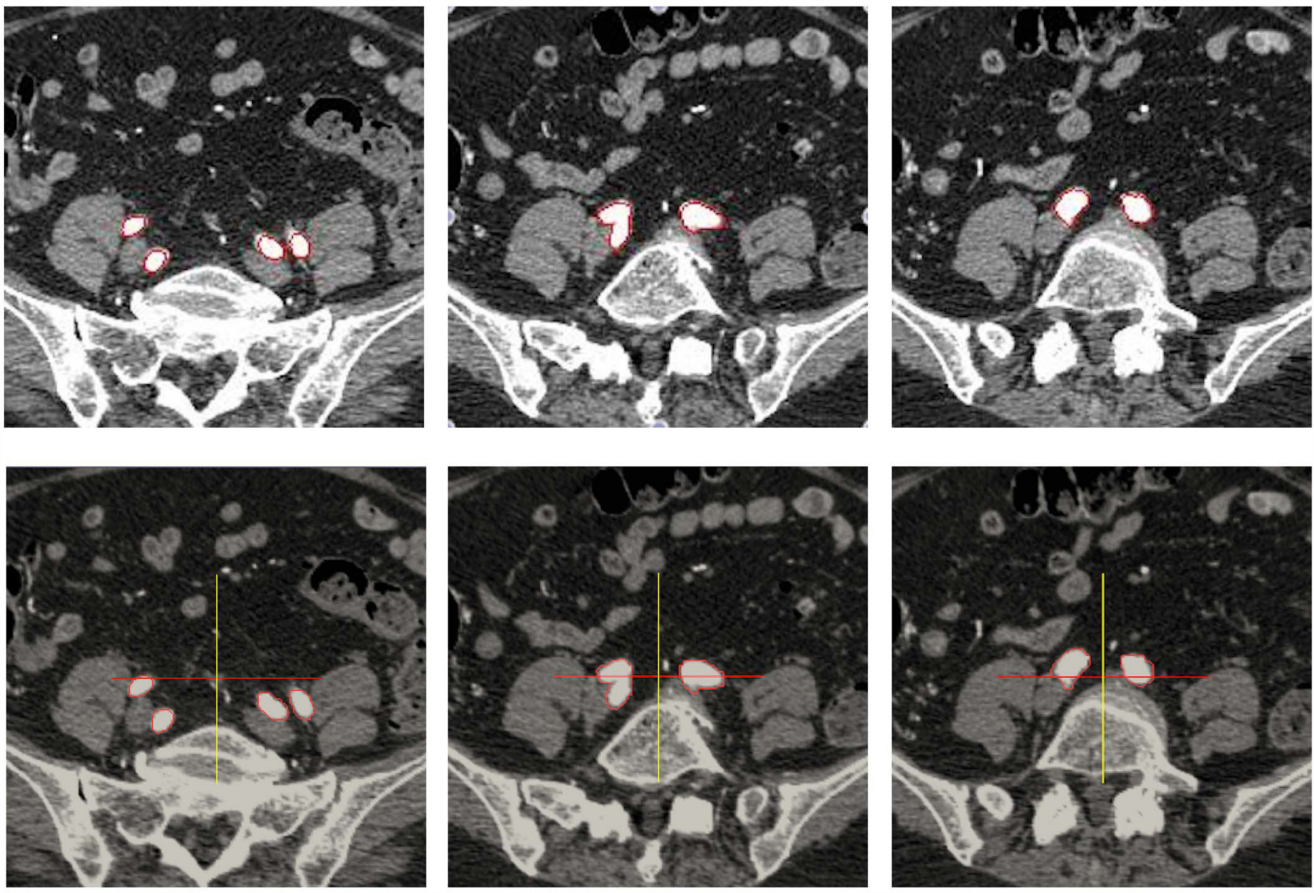
Medical data processed on uniform grids (top row) and on adaptive grids (bottom row) illustrating the similarity in results. In both cases, 268 frames with size 202 × 201 are processed. Timing is 25 minutes (1500 seconds) in the case of uniform grids and about 3.5 minutes (216 seconds) in the case of the current approach on adaptive grids.

**Fig 7:**
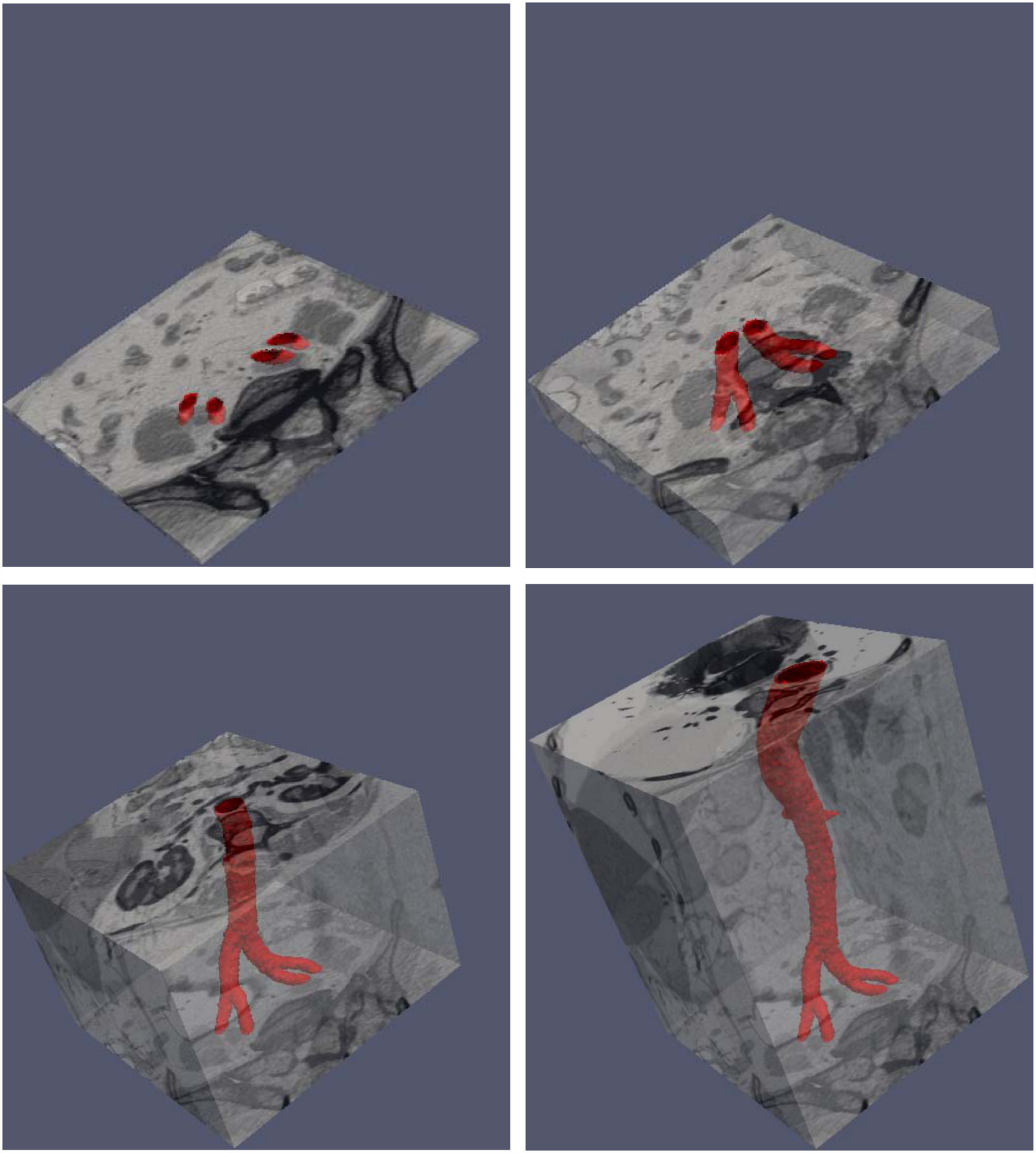
Three dimensional representation of the segmentation of Fig 6, which illustrates the changes in topology.

We consider a medical data sequence where the objects of interest are vessels. In the first slice, the objects of interest are four cross-sections of vessels, which merge into two larger vessels and then into one large vessel in subsequent slices. This example illustrates the error-free tracking effectiveness. Fig 6 compares the results obtained with the approach of Ghosh *et al.* [16] (top subfigures) with the results of the current approach (bottom subfigures). These results demonstrate that the four initial vessels are indeed properly detected and that the algorithm enables changes in topology (here merging). Fig 7 depicts the three-dimensional segmentation obtained from the series of two-dimensional segmentations.

The use of the adaptive grid significantly reduces the number of pixels processed. Specifically, the uniform grid contains 201 × 201 = 40, 401 cells, whereas the adaptive grid processes about 1000 cells at each iteration. Therefore, even though the adaptive approach requires the data structure’s management, the remeshing at each step and the *O*(log(*n*)) complexity for accessing data (*n* being the number of cells processed), the adaptive approach increases the speed in a meaningful way: on this example, the process time is more than 25 minutes on the uniformly sampled image, compared to less than 5 minutes with the same parameters and the same number of frames in the case of adaptive grids. We note that no attempts has been made to optimize the implementation, for which, in average, the overhead time for considering the adaptive grid is about 0.03 second per slice. This amounts to 7.71 seconds over the course of the total segmentation that takes a total computational time of 201.66 seconds. For this example, the overhead for using adaptive grids is thus about 4%.

### 5.3 Three Dimensional Data

We consider three different sequences of three dimensional medical data consisting of snapshots of a beating heart, available from the BIRN data base [35]. Each frame has dimensions 100 × 100 × 34. The sequences, corresponding to patient 12, 13 and 15 consist of 20, 21 and 12 frames, respectively.

**Fig 8:**
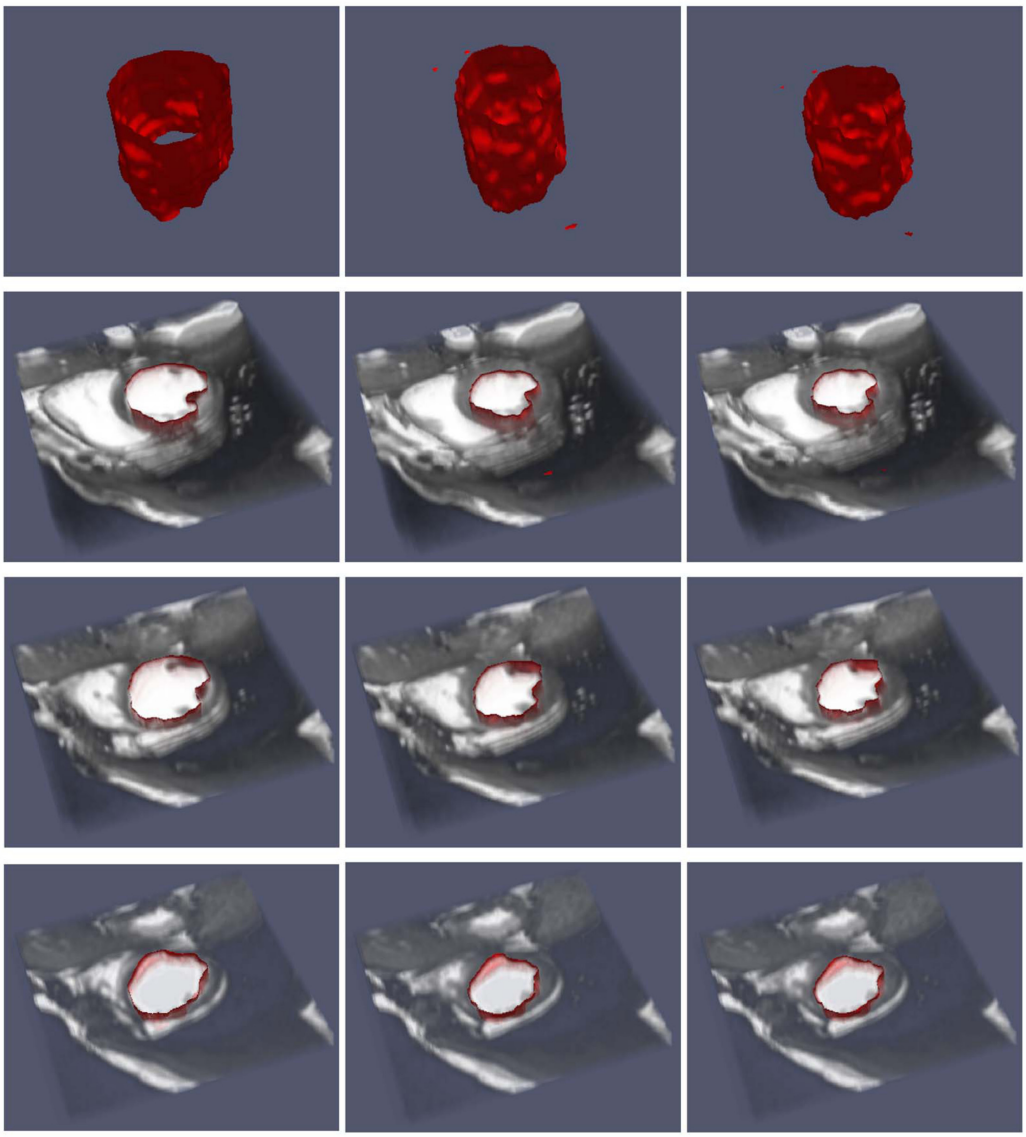
Image sequence for a half period (T/2) for patient 12. The top row gives the segmentation in 3D, while the remaining rows give the top, middle and bottom cross-sections of the segmentation on top of the data for *t* = 0, *t* = *T*/4 and *t* = *T*/2, respectively.

**Fig 9:**
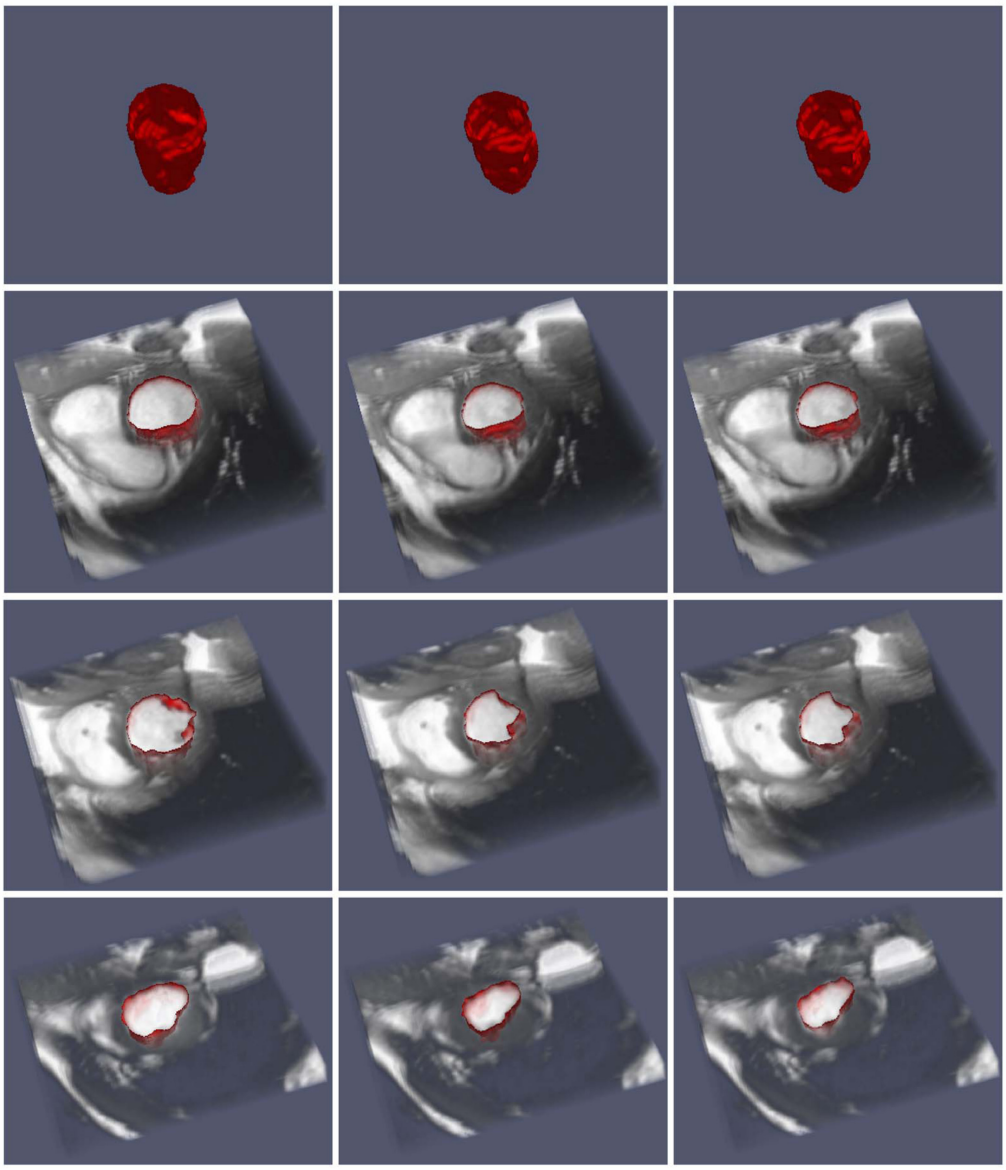
Image sequence for a half period (T/2) for patient 13. The top row gives the segmentation in 3D, while the remaining rows give cross-sections of the segmentation on top of the data.

**Fig 10:**
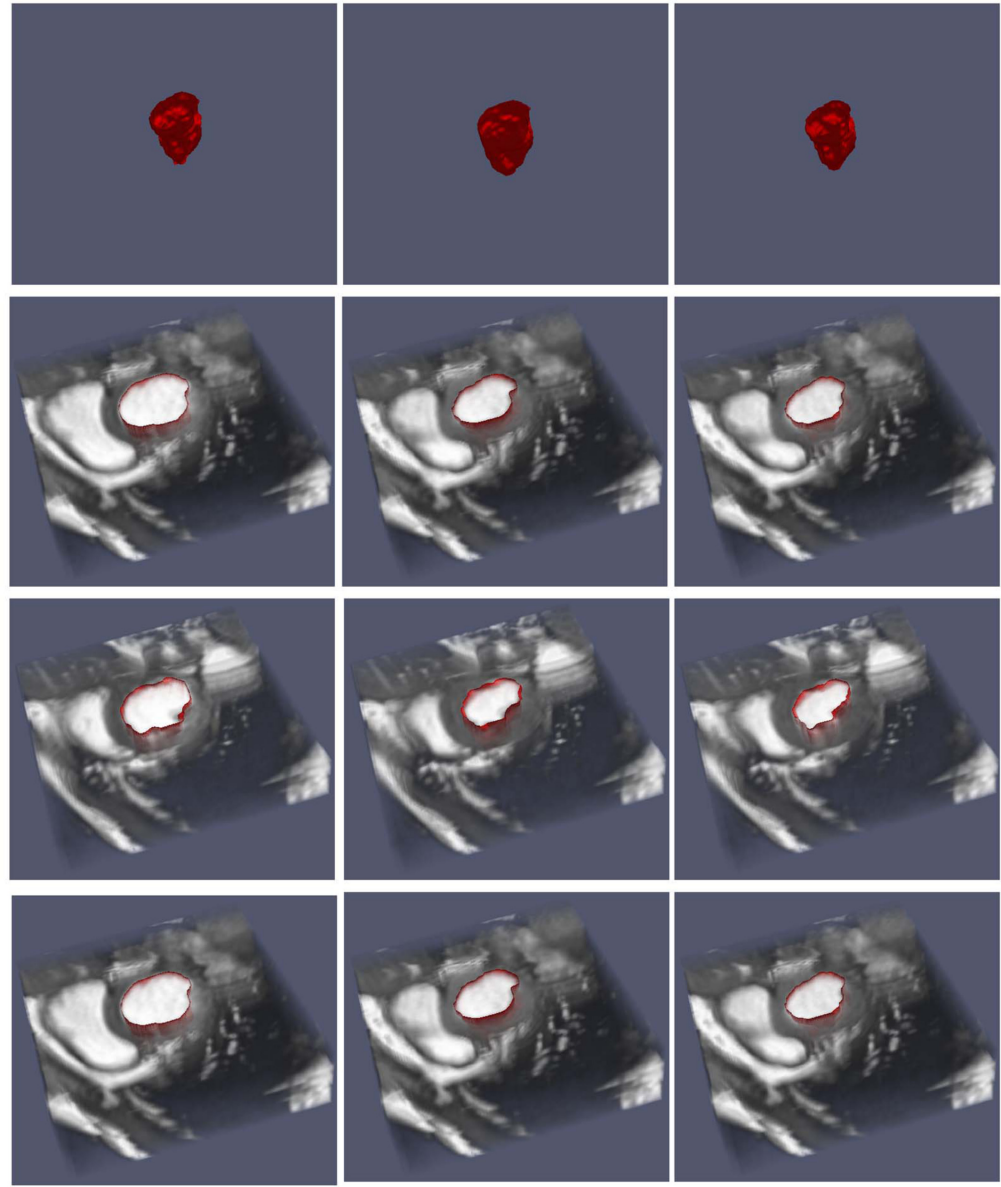
Image sequence for a half period (T/2) for patient 15. The top row gives the segmentation in 3D, while the remaining rows give cross-sections of the segmentation on top of the data.

Figs 8, 9 and 10 depict the results of the segmentation process using the current approach. In each case, three different cross sections (corresponding to a top, medium and bottom cross-section) are depicted at three different time in the image sequence. In all cases, the segmentation results are satisfactory. We note that since the algorithm requires an initial segmentation, we used one 2D cross-section of the first frame and applied the 2D algorithm to construct the initial 3D segmentation. Therefore, overall, only an initial 2D segmentation is needed to process the time sequence of three dimensional data. The algorithm takes about 55 seconds to process one 3D frame with the Octree representation and about 560 seconds for the uniform one.

### 5.4 Large Deformation and Disconnected Sets

**Fig 11:**
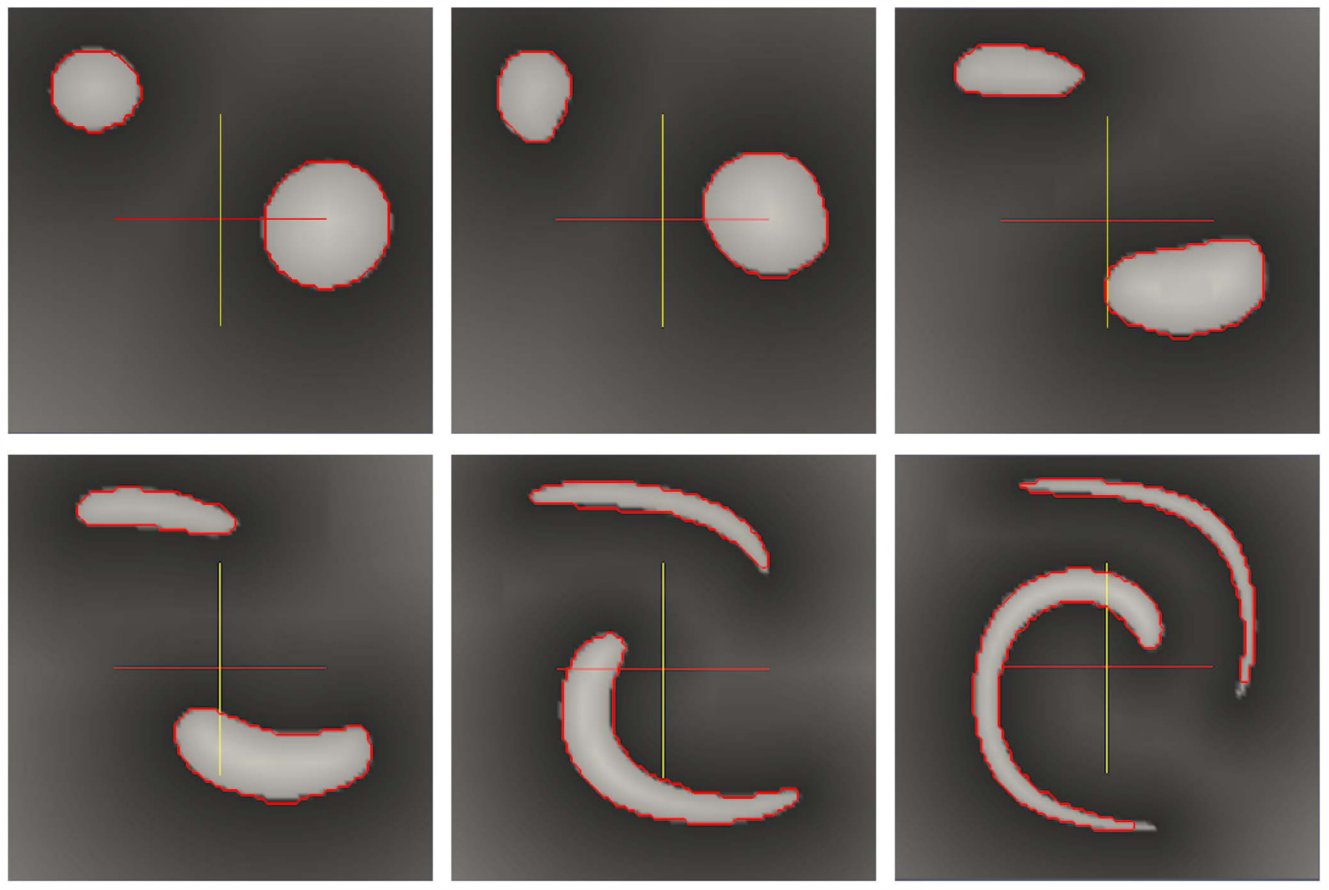
Two vortex in 2D: 500 frames with size 100 × 100, performed in 637.65 seconds.

We consider a synthetic example to demonstrate that the method is applicable to the case where the shape of interest undergoes large deformations. It also demonstrates that disconnected sets can be readily considered. We first built an image sequence consisting of two disks deforming under a given velocity field: in a domain Ω = [0, 1]^2^, we construct a disk of radius.15 and center (.5,.75) and a second disk with a radius of.1 and center (.2,.2). These two disks define the initial zero level set contour. The image intensity values are then randomly set to be between [200, 255] inside the zero level set with a value of 200 near the level set contour and between [50, 155] outside the zero level-set with a value of 50 near the contour. The image is then deformed under the divergence free velocity field **u** = (*u*, *v*) given by:

$$\begin{array}{ll} u(x,y)\;= & -{\text{sin}}^{2}(\pi x)\text{sin}(2\pi y),\\ v(x,y)\;= & {\text{sin}}^{2}(\pi y)\text{sin}(2\pi x), \end{array}$$

and the algorithm on quadtree is used to segment the image sequence. Fig 11 illustrates that the algorithm on adaptive grids enables the segmentation of a disconnected shape that undergoes large deformations. The computational time on this 100 × 100 image is slightly more than a second per frame.

## 6 Conclusion

We have extended the approach of [17] to the case of adaptive Cartesian grids using Quad-/Oc-tree data structures. This approach is based on a robust variational framework for level-set-based simultaneous registration and segmentation procedures that can be applied to natural and biological image sequences. In addition, the use of adaptive grids produces results that can be processed much faster than [17] while retaining its accuracy and robustness. Using numerical results on image sequences in two and three spatial dimensions as well as timings, we have illustrated the computational efficiency of the approach. While we have presented the use of adaptive grids using a particular functional, the advantages of adaptive grids (reduction in the number of processed data and the ability to focus on processing data near the moving front) could be used with other functionals used in level-set formulations. The strategy presented in this paper could be use to the segmentation of large data sets, such as those generated by new generation CT acquisition scanners or by structural MRI scans.
